# Characterisation of human papillomavirus (HPV) genotypes in the Azorean population, Terceira island

**DOI:** 10.1186/1750-9378-3-6

**Published:** 2008-04-21

**Authors:** Isa Dutra, Margarida R Santos, Marta Soares, Ana R Couto, Maria Bruges-Armas, Fernando Teixeira, Luísa Monjardino, Shirley Hodgson, Jácome Bruges-Armas

**Affiliations:** 1 Serviço Especializado de Epidemiologia e Biologia Molecular (SEEBMO), Hospital de Santo Espírito de Angra do Heroísmo, Portugal; 2 IBMC-Instituto de Biologia Molecular e Celular da Universidade do Porto, Portugal; 3 Serviço de Obstetrícia e Ginecologia, Hospital de Santo Espírito de Angra do Heroísmo, Portugal; 4 Cancer Genetics, St George's University of London, UK

## Abstract

**Background:**

Human papillomavirus detection is very important for the evaluation of prevention strategies in cervical cancer. In the Azorean population, the virus prevalence has never been studied, and there is no data available to preview a successful outcome with HPV vaccination. In this article, our objective is to characterise the HPV genotypes in Terceira Island, contributing for the epidemiological knowledge on the virus infection.

**Results:**

Cervical samples were collected from 289 women aged 16–81 in the Gynaecological Outpatient Clinic of the Hospital de Santo Espírito de Angra do Heroísmo (HSEAH). HPV DNA was amplified by Polymerase Chain Reaction using the general consensus primers PGMYO9/PGMY11. Commercially available Papillomavirus Clinical Arrays^® ^kits (Genomica) were used to perform HPV genotyping. 30 women were HPV positive, with a median age of 41 years old. Our results show that the overall HPV prevalence was 10.49%. Seventeen genotypes were identified, including 58.82% high risk, 17.65% low risk and 23.53% undetermined risk.

**Conclusion:**

Unlike other epidemiological studies, HPV31 was the most frequent type (26.67%) in Terceira Island, followed by HPV16 (10.00%), HPV51, HPV53, HPV70 and HPV82 (6.67%). Further studies are needed to investigate if the HPV types found in our population are associated with the risk of progression to high-grade squamous intraepithelial lesions or cervical cancer.

## Background

Human Papillomavirus (HPV) is the main responsible of one of the most common sexually transmitted diseases worldwide and persistent infection is the major risk factor for the development of cervical cancer.

To date, more than one hundred genotypes have been identified and 40 types are sexually transmitted and infect the cervix [1,2]. Most cervical infections are transient and cause either no detectable or mild pathological changes, but in some instances, infections persist and can progress over the course of several years to cervical intraepithelial neoplasia (CIN), and then possibly to invasive cervical cancer.

Detailed epidemiological studies of HPV infection, CIN and progression to cancer have tailored which are the most frequent types playing a key role in cervical carcinogenesis (high risk types), and optimal strategies are been designed to prevent, via HPV screening and vaccination, the approximately 200,000 deaths caused by this disease annually worldwide [1].

Epidemiological studies on the prevalence of HPV types in cervical cancer show that 50% of all cases are related to HPV 16. The others are associated mainly with HPVs 18, 45, 31, 33, 52, 58 and 35, including infections with both single and multiple types [3].

In the European Union, there were over two million incident cases of cancer in 2006 and over one million cancer deaths. In women, breast cancer was the most common form of cancer, followed by colorectal cancer and there were 82 500 (8.0%) cases of cervical cancer [4]. Portugal has a relatively high incidence of cervical cancer in the European Union, and the high frequency of HPV-16 infection is in concordance with other white populations [5].

Cervical cancer is largely preventable through cytological screening programs designed to facilitate the detection and treatment of immediate precancerous lesions. These evaluations require trained cytotechnologists and up to three visits for screening [6]. Alternative methods such as DNA testing for human papillomavirus, especially high-risk types, are becoming increasingly attractive as a primary screening tool, because of sensitivity and cost-effectiveness [7].

Despite the fact that prevalent HPV types are almost the same in all regions of the world, it is important to improve HPV genotype characterisation worldwide, including under-reported regions such as the population of the Azores, helping to define preventive strategies better.

The aim of this study is to participate in the global effort to characterise HPV genotypes, contributing for the fulfilment on the epidemiology of HPV infection.

## Methods

### Study population

A total of 289 women with a median age of 41 years (range 16 to 81 years) participated in this study between February 2006 and January 2007. The population was consecutively recruited from the Gynaecology Outpatient Clinic at the Department of Obstetrics and Gynaecology of the Hospital de Santo Espírito de Angra do Heroísmo (HSEAH). The Ethics Committee of Hospital de Santo Espírito de Angra do Heroísmo, approved the study protocol.

#### Sample collection

Sample material was rinsed into a liquid medium (PreservCyt, Cytyc Corporation), transported to Serviço Especializado de Epidemiologia e Biologia Molecular (SEEBMO) of HSEAH and stored at 4°C before DNA extraction.

### DNA extraction and purification

The DNA from cervical samples was extracted and purified, using Papillomavirus Clinical Arrays^® ^(Genomica) kit, according to the manufacturer's instructions. Briefly, after 1 ml cell suspension centrifugation (10 minutes, 12 000 rpm, room temperature), the lysis buffer and the proteinase K (20 mg/ml) were added on the pelleted cells and incubated 2–3 hours ate 56°C in a Thermomixer Comfort Eppendorf. After 10 minutes proteinase K inactivation period at 70°C, DNA purification was performed, using DNA purification columns and the pelleted DNA was resuspended in 100 μl Elution Buffer.

### Polymerase chain reaction (PCR)

The amplification reaction was carried out in a total volume of 50 μl, containing 5 μl of extracted DNA and 45 μl of a PCR mix with PGMYO9/PGMY11 generic consensus primers. The PCR mix contains, beside the generic consensus primers, two internal controls that will validate each sample genotyping: genomic DNA control and amplification control. The first control consists in a primer set that amplifies a 892 bp CFTR gene fragment and the second control amplifies 1202 bp of a modified plasmid, using the same primers as the CFTR gene.

The mixture was submitted to 45 cycles of amplification, using a DNA thermalcycler T-Gradient (Biometra). Each cycle included a denaturation step at 94°C for 30 seconds, followed by an annealing step at 55°C for 1 minute and a chain elongation step at 72°C for 90 seconds. Each PCR was initiated with a 9 minutes denaturation step at 95°C and finished by an 8 minutes extension-step at 72°C.

Different laboratory areas were used for sample handling (pre-PCR area), product amplification (PCR area) and HPV genotyping (post-PCR area).

### HPV testing

The commercially available Papillomavirus Clinical Arrays^® ^(Genomica) kit was used for HPV DNA genotyping. All samples were analysed for the presence of he following HPV types: 6, 11, 16, 18, 26, 31, 33, 35, 39, 40, 42, 43, 44, 45, 51, 52, 53, 54, 56, 58, 59, 61, 62, 66, 68, 70, 71, 72, 73, 81, 82, 83, 84, 85 and 89.

HPV detection was conducted using PGMYO9/PGMY11 general consensus primers designed to amplify a 450 bp HPV L1 gene fragment. This region is used because it is highly conserved between different HPV types but has sufficient variation for the identification of each one [2].

The detection of the amplified PCR product was performed with a new technological platform using a low density Microarray, anchored in 2 ml tube-AT tube. It allows simultaneous detection of multiple molecular markers in the L1 fragment of 35 different HPV types and in the necessary controls to insure a feasible assay. All process was followed according to the manufacture's standard protocol.

PCR products were marked with biotin and, after amplification, they hybridised with the respective probes in specific known AT tube areas. Biotin binds to streptavidin-peroxidase after incubation. The addition of the TMB substrate (3,3',5,5'-tetrametilbinzidine) generates an insoluble product after hybridisation.

The results were processed by software, which allows detection, interpretation and reporting for each sample.

## Results

The study encompasses two hundred and eighty nine women with a mean age of 41 years and was stratified in 5-year age groups.

Two hundred and seventy five (95.2%) women had normal cytology. Epithelial cell abnormalities were detected in 14 women: 4 were labelled as atypical squamous cell of undetermined significance (ASCUS; 1.38%), 7 had cervical intraepithelial neoplasia (CIN; 2.42%), and 3 women (1.04%) had been treated for cervical carcinoma and were excluded from the study sample. The three exclusion cases were HPV positive: HPV16, HPV61 and 66 and HPV58.

HPV-DNA was detected in thirty (10.49%) out of tow hundred and eighty six eligible women and only one had cytological abnormalities (CIN).

**Figure 1:**
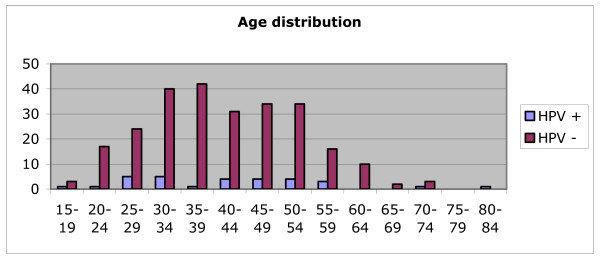
Age-distribution in HPV-positive and HPV-negative women.

Figure 1 shows the age distribution of population under investigation, discriminating HPV positive and negative women; two peaks in age groups 25 to 34 and in 40 to 54 years old are clearly in evidence. Women younger than 25 years and older than 59 years had a lower HPV prevalence.

Seventeen HPV types were detected, encompassing three low-risk types (17.65%) – including a double low risk infection, ten high-risk types (58.82%), and four undetermined risk types (23.53%).

Single infections were found in 26 women (86.67%) and multiple infections were detected in 4 (13.33%), ranging from 2 to 3 different genotypes (average of 1.17 types per women).

**Figure 2:**
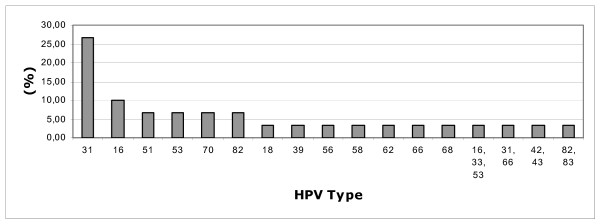
Human Papilomavirus type distribution.

The most common types of Human Papillomavirus in Terceira Island were HPV31, followed by HPV16, HPV51, HPV 53, HPV70 and HPV82 (Figure 2).

## Discussion

In the present study, we intended to evaluate the prevalence of different HPV type infection in a sample of women from the Azorean population. Among those referred to a Gynaecologic Outpatient Clinic, the prevalence of HPV infection in the Azorean population (10.49%) was concordant with the range of 2 to 44% world-wide [8].

Burchell et al showed that age-specific HPV prevalence among women with normal cytology was highest for younger women (<20) and decreased in the middle age groups, to increase again at age 65 and older [9]. This finding is not concordant with our results were HPV prevalence was highest between 25 and 34 years old and in middle age groups (40 to 54 years old) (Figure 1). Regarding older women, there were no women on age groups 75 to 79 years old, but HPV infection decreased from 60 to 84 years old in our sample.

The second major peak of infection identified in our population, as described in other studies, was in the peri- or post-menopausal years. Although the reason for this "menopausal peak" is still unclear, it may be related to the reactivation of latent infections acquired earlier in life, due to a gradual loss of type-specific immunity or to acquisition of new infections by sexual contacts with new partners later in life [8,9].

The most common HPV types in Clifford et al meta-analysis, in either single and multiple infections, were HPV16, HPV 42, HPV 58, HPV 31, HPV 18, HPV 56, HPV 81, HPV 35, HPV 33 and HPV 45 [7].

In our sample, the majority of HPV-positive women were infected with the high-risk HPV 31 – 26.67% (Fig. 2), which has a distribution of 9%, 7%, 5% and 4% in Europe, Sub-Saharan Africa, South America and Asia, respectively, according to the Clifford study.

Rates of HPV-16 infected women in Azores (10.00%) were quite similar to those in Asia (14%) and much lower than in Europe (21%).

HPV18 infection among women was very similar across all world regions, varying from 4 to 5%; slightly lower results were obtained in the Azores population (3.33%).

The discrepancy between our data and previous European publications may be related to important influxes of groups coming from Europe; especially Eastern Europe (Ukraine and Russia), Africa (Cabo Verde), Latin America and North America.

Prevention of HPV infection through vaccination is expected to dramatically reduce the morbidity and mortality associated with HPV infection. These vaccines are based on the major capsid protein of the virus, L1 proteins, which are capable of self-assembling into virus like particles (VLPs) when expressed in cells. These VLPs share great similarity to native HPV virions, are non-infectious and non-oncogenic and can induce high levels of neutralizing antibodies [10,11].

Two vaccines are currently available and they incorporate HPV types more frequently associated with cervical cancer. A bivalent VLP vaccine (Cervarix™ GlaxoSmithKline) is composed of the assembled VLPs of HPV16 and HPV18 L1, and a quadrivalent VLP based vaccine (Gardasil^® ^Merck & Co) that includes HPV16, HPV18, HPV6 and HPV11; these last two types cause the majority of genital warts (approximately 90%) in both men and women [10].

The proportions of high-risk HPV infections preventable by a vaccine for HPV16 and HPV18 vary by region, being highest in Europe and lowest in Sub-Saharan Africa [12]. However, the available vaccines do not contain HPV31, which is prevalent in the Azorean population.

Determining whether any significant protection is provided between genotypes will be a prerequisite to increase the breadth of genotype coverage for prophylactic vaccines. If VLP vaccines are found to confer a high rate of type-specific protection but no significant cross-protection, alternative second-generation vaccines may be needed to protect against other high risk HPV types, such as HPV45 and HPV31, to perform a major reduction in cervical cancer [13].

## Conclusion

Future studies are necessary to confirm some study issues. It is extremely important to enlarge our sample in order to confirm the very high HPV31 prevalence not only in normal cytology but also in high-grade lesions and in cervical cancer.

These findings will let us know about the efficacy of the existing vaccines and if a new one should be tailored for the population under investigation.

## Competing interests

The authors declare that they have no competing interests.

## Authors' contributions

MBA, FT and LM carried out cervical sample collection. MS, MRS and ARC carried out DNA extraction. ID carried out DNA extraction, HPV genotyping, and drafted the manuscript. SH reviewed the manuscript. JBA coordinated and reviewed manuscript. All authors read and approved the final manuscript.
